# Antioxidant, Antinociceptive and Anti-inflammatory Activities of Ethanolic Extract of Leaves of *Alocasia indica* (Schott.)

**DOI:** 10.4103/0975-1483.63152

**Published:** 2010

**Authors:** WA Mulla, SB Kuchekar, VS Thorat, AR Chopade, BS Kuchekar

**Affiliations:** *Government College of Pharmacy, Vidyanagar, Karad 415 124, India*; 1 *Allana College of Pharmacy, Pune-411 001, India*; 2 *Rajarambapu College of Pharmacy, Kasegaon, Taluka, Walwa District, Sangli- 415 404, Maharashtra, India*; 3 *MAEER’s Maharashtra Institute of Pharmacy, Kothrud, Pune-411 038, India*

**Keywords:** *Alocasia indica* Schott, antioxidant, antinociceptive, anti-inflammatory, ascorbic acid, diclofenac

## Abstract

Extracts obtained from the leaves of various *Alocasia* species have been used in India as folk remedy for the treatment of various inflammatory ailments including rheumatism and bruise. The ethanolic extract of leaves of *Alocasia indica* Schott. was evaluated by using different *in vitro* antioxidant models of screening like scavenging of 1, 1-diphenyl-2-picryl hydrazyl (DPPH) radical, nitric oxide radical, superoxide anion radical, and hydroxyl radical. The antinociceptive activity was tested by acetic acid-induced writhing response, hot plate method, and tail flick method in albino rats. The anti-inflammatory potential of gels of ethanolic extract has been determined by using carrageenan-induced paw edema assay, formalin-induced paw edema assay, arachidonic acid-induced ear edema assay, and xylene-induced ear edema assay. The extract showed remarkable antioxidant activity in all models, comparable to the standard reference drug ascorbic acid. The ethanolic extract of *Alocasia indica* and its gels produced dose-dependent antinociceptive and anti-inflammatory activity, respectively. This finding suggests that ethanolic extract of *A. indica* possess potent antinociceptive and anti-inflammatory activity possibly due to its free radical scavenging properties.

## INTRODUCTION

*Alocasia indica* Schott. is an indigenous herb belonging to family Araceae. Different parts of this plant are traditionally used in inflammation and in diseases of abdomen and spleen.[1] The juice of leaves of the plant is used as digestive, laxative, diuretic, astringent, and for the treatment of rheumatic arthritis.[2] It has hepatoprotective[3] and antifungal properties.[4] This plant contains flavonoids, cynogenetic glycosides, ascorbic acid, gallic acid, mallic acid, oxalic acid, alocasin, amino acids, succinic acid, and β-lectines.[5] Since no scientific data are available to justify the traditional anti-inflammatory potential of the plant, the present study was planned to validate the therapeutic use of this plant in treatment of inflammatory conditions.

## MATERIALS AND METHODS

### Chemicals

All chemicals and solvents were of analytical grade and obtained from Loba Chemicals Ltd, Mumbai. 1, 1-diphenyl, 2-picryl hydrazyl (DPPH) was obtained from Sigma Chemicals, USA. The other chemicals used were sodium nitroprusside, sulphanilamide, *O*-phosphoric acid, napthyl ethylene diamine dihydrochloride, trichloroacetic acid (TCA), glacial acetic acid, nitroblue tetrazolium (NBT), ethylene diamine tetra acetic acid (EDTA), riboflavin, and Fe-EDTA. Carrageenan was obtained from Sigma-Aldrich, USA. Volini gel (Diclofenac sodium) was a gift sample from Ranbaxy Laboratories Ltd, Punjab. UV-Visible spectrophotometer (Shimadzu 1700) was used for recording the spectra.

### Experimental animals

Wistar albino rats weighing 175-225 g of either sex were obtained from Krishna Institute of Medical Sciences, Karad, Dist- Satara (Maharashtra) and were acclimatized for 10 days under standard housing conditions (24° ± 1°C; 45-55% RH with 12:12 h light/dark cycle). The animals had free access to rat food (Lipton Gold Mohr, India) and water. The animals were habituated to laboratory conditions for 48 h prior to the experimental protocol to minimize any nonspecific stress. The experimental protocol was approved by the Institutional Animal Ethics Committee by Government College of Pharmacy, Karad, India, and animals were maintained under standard conditions in the animal house approved by Committee for the Purpose of Control and Supervision on Experiments on Animals (CPCSEA).

### Plant material

Fresh leaves of *A. indica* Schott. (Araceae) collected from different places at Karad were authenticated at Botany Dept., Yashwantrao Chavan College of Science, Karad, and a voucher specimen was deposited at the Herbarium of institute.

### Preparation of extract

The ethanolic extract of leaves of *A. indica* was prepared by soxhletion. The powdered plant material (750 g) was repeatedly extracted in a round bottom flask with 2250 ml ethanol. The reflux time was 40 cycles for complete extraction. The extract was cooled at room temperature, filtered, and evaporated to dryness under reduced pressure in a rotary evaporator. The percentage yield was found to be 2.79 g. The ethanolic extract of *A. indica* was referred as AI.

### Phytochemical screening

Chemical tests were carried out on ethanolic extract of the plant for qualitative determination of phytochemical constituents.[6]

### *In vitro* antioxidant activity

*DPPH assay:* To 1 ml extract of different concentrations, 1 ml solution of DPPH (0.1 mM) was added. An equal amount of methanol and DPPH solution served as control. After 20 min of incubation in the dark, absorbance was measured at 517 nm.[7,8] Ascorbic acid was used as standard. The experiment was performed in triplicate and the percentage scavenging was calculated.

*Scavenging of nitric oxide radical:* Nitric oxide was generated from sodium nitroprusside and measured by Griss reaction.[9,10] Sodium nitroprusside (5 mM) in standard phosphate buffer saline solution (0.025 M, pH 7.4) was incubated with different concentrations of (200-1000 µg/ml) of the ethanolic extract dissolved in phosphate buffer saline (0.025 M, pH 7.4) and the tubes were incubated at 25 °C for 5 h. Control experiments without test compounds but with equivalent amount of buffer were conducted in identical manner. After 5 h, 0.5 ml of solution was removed and diluted with 0.5 ml of Griss reagent (2 g of 1% sulphanilamide, 5 ml of 2% *O*-phosphoric acid, and 2 g of 0.1% napthyl ethylene diamine dihydrochloride). The absorbance of chromophore formed during diazotization of nitrite with sulphanilamide and its subsequent coupling with napthyl ethylene diamine was read at 546 nm. Ascorbic acid was used as standard. The experiment was performed in triplicate.

*Hydroxyl radical scavenging activity:* Extracts of different concentrations (200-1000 µg/ml) were taken in different test tubes and evaporated on water bath. To these, 1 ml of Fe-EDTA, 0.5 ml of EDTA, and 1 ml DMSO were added and the reaction was initiated by adding 0.5 ml ascorbic acid to each of the test tubes. Test tubes were capped tightly and heated in water bath at 80-90 °C for 15 m. Then the reaction was terminated by addition of 1 ml of ice-cold TCA (17.5%, w/v) to all the test tubes and kept aside for 5 min. The formaldehyde formed was determined by adding 3 ml Nash reagent (75 g ammonium acetate, 3ml glacial acetic acid, 2 ml acetyl acetone was mixed and raised to 1 l with distilled water). This reaction mixture was kept aside for 15 min for color development.[11] Intensity of yellow color formed was measured spectrophotometrically at 412 nm against reagent blank. Ascorbic acid was used as standard. Percentage scavenging was calculated by comparison of the result of the samples, standard, with that of the blank.[12]

*Scavenging of superoxide radical by riboflavin--NBT system:* The assay was based on the capacity of the sample to inhibit blue formazon formation by scavenging the superoxide radicals generated in the riboflavin--NBT system. The reaction mixture contains 50 mM phosphate buffer pH 7.6, 20 g riboflavin, 12 mM NBT. Reaction was started by illuminating the test samples of the extract (200-1000 µg/ml). The absorbance was measured at 590 nm. Ascorbic acid was used as positive control.[13,14]

The percentage scavenging of each extract in different concentrations for different *in vitro* models are calculated by following formula:

$$\%\mathrm{Scavenging}\;=\;\frac{\mathrm{Absorbance}\;\mathrm{of}\;\mathrm{standard}\;-\;\mathrm{absorbance}\;\mathrm{of}\;\mathrm{extract}\;\times \;100}{\mathrm{Absorbance}\;\mathrm{of}\;\mathrm{standard}}$$

IC_50_ was calculated by using formulae:

*b* = ∑ *x.y* / ∑*x^2^*

*a* = *y* − *bx*

IC_50_ = *a* + *b* (50)

Where, *b* = regression coefficient of *x* on *y*; *a* = intercept of the line; *x* = concentration; and y = % scavenging; x = mean of the concentration; y = mean of % scavenging.

### Antinociceptive activity

*Writhing test:* Writhing was induced in rat (n = 6) by intraperitoneal injection (10 ml/kg) of 0.6% acetic acid. The number of writhings was counted over a 20 min period as previously reported.[15,16] Animals were treated through oral route 30 min before injection of acetic acid with ethanolic extract of AI (200 and 400 mg/kg) or acetylsalicilic acid (200 mg/kg). The control group received only vehicle (3 ml/kg) of 1% suspension of Tween-80.

*Tail immersion test:* Six rats were administered orally with vehicle (3 ml/kg), pentazocine (30 mg/kg), and ethanolic extract of AI (200 and 400 mg/kg). The distal part of the tails of the animals was immersed in hot water maintained at 55.0 ± 1.0 °C. The time taken to withdraw the tail was noted as reaction time.[17] A cut-off time of 10 s was maintained at 55 °C to prevent tissue damage. The reaction time was measured at 0, 15, 30, 45, and 60 min after treatment, respectively.

*Hot plate test:* Rats were placed on an aluminum hot plate kept at 55 ± 0.5 °C for a maximum time of 30 s.[18] Reaction time was recorded when the animals licked their fore- and hind paws and jumped; at before (0) and 15, 30, 45, and 60 min after intraperitoneal administration of 200 and 400 mg/kg of ethanolic extract of AI to different groups of six animals each. Morphine 10 mg/kg was used as the reference drugs.

### Anti-inflammatory activity

*Preparation of gels:* The gels of AI were prepared with carbopol-940 and PEG by cold method.[19] Different gels of AI (5%, 10%, and 20%) were formulated [Table 1]. The weighed amount of carbopol was placed in a beaker, and sufficient amount of water was added to it and kept in oven at 100°C for 20 min to obtain a homogenous viscous mixture and cooled to room temperature with continuous stirring. Triethanolamine was added drop-wise with constant stirring. After formation of gel, weighed amount of AI was mixed in it.

**Table 1:** Formulation of gels of ethanolic extract of *Alocasia indica*

Ingredients (g)	Formulations (15 g)		
	5%	10%	20%
Ethanolic extract of Alocasiaindica	0.75	1.50	3.00
carbopol-940	0.15	0.15	0.15
*PEG-4000	0.75	0.75	0.75
PEG-200	0.75	0.75	0.75
Ethanol	3.00	3.00	3.00
Triethanolamine	0.40	0.40	0.40
Methyl paraben	0.05	0.05	0.05
Propyl paraben	0.02	0.02	0.02
Distilled water	9.20	8.40	7.70

* PEG: Polyethylene glycol.

*Carrageenan-induced paw edema assay:* The animals (n = 6) were divided into five groups. 0.5 g of AI gels (5%, 10% and 20%) and marketed formulation of diclofenac sodium (2%) were applied to the planter surface of the left hind paw by gently rubbing 50 times with the index finger.[20] The gel base without drug was applied in control group of rats by the same mode of application. After 2 h, the dose 0.1 ml of 1% carrageenan suspension (in sterile normal saline) was injected subplantarly into treated and control groups of rats. The paw volume was measured plethysmometrically (Ugo Basile, Italy) at 0 and 3 h after the carrageenan injection. The difference between the two readings was taken as the volume of edema, and the percentage anti-inflammatory activity was calculated using following equation:

Percentage anti-inflammatory activity = (*V* – *Vi/Vi*) ×100, where *V* is the paw volume 3 h after the carrageenan injection and *Vi* is the initial paw volume.

*Formalin-induced paw edema assay:* The same procedure as mentioned above in carrageenan-induced paw edema assay was followed except that acute inflammation was produced by administration of 20 µl formalin (2.5% in distilled water) into the subplanter area of right hind paw of rat.[21]

*Arachidonic acid-induced ear edema assay:* The animals (n = 6) were divided into five group. Samples were given 1 h prior to application of arachidonic acid.[22] One hour after topical application of AI gels (0.5 g) and marketed formulation of diclofenac sodium (2%), 2% arachidonic acid dissolved in acetone (0.02 ml/ear) was applied to anterior and posterior surfaces of right ear of rat. The gel base without drug was applied in the control group of rats by the same mode of application. The ear thicknesses were measured using a dial thickness gauge before and at 1 and 3 h after arachidonic acid treatment, and the differences in the thickness were calculated. The degree of ear swelling was expressed as an increase in ear thickness in mm.

*Xylene-induced ear edema assay:* The same procedure as mentioned above in arachidonic acid-induced ear edema assay was followed except that acute inflammation was produced by applying xylene to anterior and posterior surfaces of right ear of rat.[23]

### Statistical analysis

The statistical significance was assessed using one way of variance (ANOVA) followed by Bonferrini’s multiple comparison tests. The values are expressed as mean ± SE and *P* < 0.05 was considered significant.

## RESULTS

### Preliminary phytochemical investigation

The preliminary phytochemical investigation of the ethanolic extract of *A. indica* showed that it contains flavonoids, cynogenetic glycosides, citric acid, ascorbic acid, and polyphenolic compounds.

### Antioxidant activity

Five concentrations ranging from 200 to 1000 µg/ml of the ethanolic extract of *A. indica* were tested for their antioxidant potential using different *in vitro* models. It was observed that free radicals were scavenged by test compounds at different concentrations. The maximum inhibitory concentration (IC_50_) in all models viz., DPPH, nitric oxide radical, superoxide, hydroxyl radical scavenging activity, were found to be 7.30, 10.97, 9.8, and 7.86 µg/ml, respectively. The antioxidant model and percentage scavenging of each concentration of extract and standard are shown in Table 2.

**Table 2:** Antioxidant potential of ethanolic extract of *A. indica*

Different antioxidant models	% Scavenging at different concentration (ug/ml) [values are mean of three replicates] AA* (μg/ml)	Test extract: *A. indica*.						
	200	50	100	200	400	800	1000	IC50
DPPH**	88.75	40.24	44.62	53.25	61.39	63.46	73.48	7.30
Nitric oxide	74.54	34.74	53.44	63.48	71.59	73.51	84.09	10.97
Hydroxyl radical	62.35	43.24	55.37	66.15	57.23	59.28	67.96	9.8
Superoxide radical	88.25	47.26	52.18	65.71	68.12	52.84	77.17	7.86

* Ascorbic acid;

** 1,1-diphenyl, 2-picryl hydrazyl.

### Antinociceptive activity

Oral administration of the ethanolic extract of AI (200 and 400 mg/kg) significantly (*P* < 0.05) reduced the number of writhings induced by acetic acid in rat [Table 3]. The activity was comparable to that of acetylsalicilic acid (200 mg/kg, p.o.) used as a reference drug. Moreover, the extract induced protection in rat in tail immersion test [Table 4] that is comparable with the standard drug pentazocine (30 mg/kg, p.o.). The results of hot plate test presented in Table 5 showed that the i.p. administration of ethanolic extract of AI at the doses of 200-400 mg/kg significantly raised the pain threshold at observation time of 45 min in comparison with control (*P* < 0.001).

**Table 3:** Effect of the ethanolic extract of leaves of *Alocasia indica* (AI) on acetic acid-induced writhing in rat

Material	Dose (mg/kg)	No. of writhings	Inhibition (%)
Control	--	45.7 ± 4.2	
Acetylsalicylic acid	200	11.8 ± 2.4*	74.17
AI	200	21.5 ± 1.9**	52.95
	400	14.5 ± 1.8*	68.27

* *P* < 0.05,

** *P* < 0.01 vs. control, [Values are mean ± SE from six animals in each group.]

**Table 4:** Effect of the ethanolic extract of leaves of *Alocasia indica* (AI) on tail immersion method in rat

Treatment	Dose (mg/kg)	Average tail withdrawing time (s)				
		0 min	15 min	30 min	45 min	60 min
Control	--	4.20 ± 0.11	4.65 ± 0.18	4.77 ± 0.17	4.77 ± 0.15	4.80 ± 0.18
Pentazocine	30	4.16 ± 0.20*	5.39 ± 0.21*	7.37 ± 0.45*	7.53 ± 0.29*	8.18 ± 0.27*
AI	200	4.25 ± 0.21*	4.53 ± 0.12**	5.27 ± 0.13*	5.35 ± 0.18**	5.27 ± 0.11**
	400	4.62 ± 0.16*	4.17 ± 0.28*	6.92 ± 0.48**	7.05 ± 0.28*	8.48 ± 0.27**

* *P* < 0.05,

** *P* < 0.01 vs. control, [Values are mean ± SE from six animals in each group.]

**Table 5:** Effect of the ethanolic extract of leaves of *A. indica* (AI) on rat submitted to the hot plate test

Treatment	Dose (mg/kg)	Time 0 (min)	Time 15 (min)	Time 30 (min)	Time 45 (min)	Time 60 (min)
Control	–	6.25 ± 0.35	6.32 ± 0.24	6.38 ± 0.31	7.15 ± 0.38	6.16 ± 0.54
Morphine	10	6.41 ± 0.35*	12.65 ± 0.48*	14.20 ± 0.36**	14.99 ± 0.81**	14.45 ± 0.56**
AI	200	5.76 ± 0.24**	10.16 ± 0.50*	13.21 ± 0.41*	14.17 ± 0.25**	12.16 ± 0.50*
	400	6.40 ± 0.48**	12.10 ± 0.31**	13.56 ± 0.73*	14.86 ± 0.72**	12.67 ± 0.78**

* *P* < 0.05,

** *P* < 0.01 vs. control, [Values are mean ± SE from six animals in each group.]

### Stability of gels

Formulations exhibited desirable property and physical stability. AI was chemically stable in these formulations during 12-month storage at 4, 25, and 48°C.

### Anti-inflammatory activity

Gels containing AI and marketed formulation of diclofenac sodium produced dose-dependent inhibition of carrageenan and formalin-induced paw edema as compared to the control (*P* < 0.05) [Table 6]. In xylene-induced mouse ear edema assay gels containing AI and marketed formulation of diclofenac sodium significantly inhibited arachidonic acid and xylene-induced ear edema in dose dependent as compared to the control (*P* < 0.05) [Table 7].

**Table 6:** Effects of the Alocasia indica gels (AI: 5%, 10%, 20%) and marketed formulation of diclofenac sodium (DS: 2%) on carrageenan-induced rat paw edema and formalin-induced paw edema

Group	Carrageenan-induced rat paw edema		Formalin-induced rat paw edema	
	Increase in paw volume (mean ± SEM) in ml	% Inhibition of paw edema	Increase in paw volume (mean ± SEM) in ml	% inhibition of paw edema
Control	0.67 ± 0.13	-	0.58 ± 0.12	-
AI (5%)	0.37 ± 0.07*	59.5	0.36 ± 0.05*	52.3
AI (10%)	0.32 ± 0.05*	66.6	0.31 ± 0.06*	58.1
AI (20%)	0.26 ± 0.03*	72.2	0.25 ± 0.02*	62.4
DS (2%)	0.20 ± 0.02*	83.1	0.19 ± 0.01**	73.7

* *P* < 0.05,

** *P* < 0.01 vs. control, [Values are mean ± SE from six animals in each group.]

**Table 7:** Effects of the *Alocasia indica* gels (AI: 5%, 10%, 20%) and marketed formulation of diclofenac sodium (DS: 2%) on arachidonic acid-induced ear edema and xylene-induced ear edema

Group	Arachidonic acid-induced ear edema		Xylene-induced ear edema	
	Increase in paw volume (mean ± SEM) in ml	% Inhibition of paw edema	Increase in paw volume (mean ± SEM) in ml	% Inhibition of paw edema
Control	0.27 ± 0.08	-	0.24 ± 0.05	-
AI (5%)	0.19 ± 0.06*	42.1	0.17 ± 0.06*	41.2
AI (10%)	0.15 ± 0.02*	45.3	0.14 ± 0.03*	44.4
AI (20%)	0.13 ± 0.03*	52.2	0.12 ± 0.03*	53.6
DS (2%)	0.09 ± 0.01**	63.5	0.08 ± 0.01**	61.1

* *P* < 0.05,

** *P* < 0.01 vs. control, [Values are mean ± SE from six animals in each group.]

## DISCUSSION

Recent studies suggest that the inflammatory tissue damages are due to the liberation of reactive oxygen species from phagocytes invading the inflammation sites.[24–26] In addition to this, nitric oxide is also implicated in inflammation, cancer, and other pathological conditions.[27] Interactions between superoxide and nitric oxide regulate the vascular tone or inflammation.[28] The significant *in vitro* antioxidant activity of AI was in a concentration-dependent manner.

The ethanolic extract of AI at the doses of 200-400 mg/kg protected rat against both chemical- and thermal-induced noxious stimuli, which were evidenced from the acetic acid-induced writhing, tail immersion, and hot plate tests. Acetic acid induces writhing syndromes[15,16] and causes analgesia by releasing of endogenous substances, which then excite the pain nerve endings; the abdominal constriction is related to the sensitization of nociceptive receptors to prostaglandins.[29] Hot plate test was also assayed to characterize the analgesic activity of extract. It is possible that ethanol extract of AI extract exerts an analgesic effect probably by inhibiting the synthesis of prostaglandins. Variation in order of activity for ethanol extract of AI fractions in acetic acid-induced writhing, tail immersion, and hot plate tests indicated that the different constituents present in different fractions may be responsible for central and peripheral analgesia.

Carrageenan-induced edema is a biphasic response in which the involvement of the cyclo-oxygenase products of arachidonic acid metabolism and the production of reactive oxygen species are well established.[30] The first phase is mediated through the release of histamine, serotonin, and kinins, whereas the second phase is related to the release of prostaglandin oxygen-derived free radicals and production of inducible cyclo-oxygenase which peak at 3h.[31] The AI extract produced dose-dependent and significant inhibition of carrageenan-induced paw edema comparable in magnitude with the inhibitory action of diclofenac. The formalin-induced paw edema assay defines distinctive biphasic nociceptive response termed neurogenic and inflammatory phases.[32] The ability of AI to have effect on both phases shows that it contains active anti-inflammatory principle acting both centrally and peripherally.

Another model of inflammation used is the ear edema induced by arachidonic acid and xylene, which serves to identify the mediators of inflammation of the lipoxygenase pathway.[33,34] Topical application of the gels of AI extract at the concentrations of 5%, 10%, and 20% inhibited the ear edema induced by arachidonic acid and xylene in dose-dependent manner similar to diclofenac. The results show that the AI acts on both the cyclo-oxygenase and lipoxygenase pathways.

Preliminary phytochemical screening showed that ethanolic extract of the leaves of *A. indica* contains flavonoid compounds. Flavonoids have been shown to possess various biological properties related to antioxidant, antinociceptive, and anti-inflammatory mechanisms by targeting reactive oxygen species and prostaglandins which are involved in the late phase of acute inflammation and pain perception.[35–37] It can be concluded that ethanolic extract of *A. indica* possess antioxidant, antinociceptive, and anti-inflammatory activities may be due to the presence of flavonoids and other polyphenolic moieties present in it, which seems to support the use of this plant in traditional medicine. Finally, the precise mechanism(s) and site(s) of this activity and the active constituent(s) involved are still to be determined in addition to toxicological studies.
